# Editorial: Radiofrequency ablation in liver cancers: Investigations of efficacy as monotherapy and polytherapy

**DOI:** 10.3389/fonc.2022.1088012

**Published:** 2022-12-13

**Authors:** Gianluca Rompianesi

**Affiliations:** Department of Clinical Medicine and Surgery, Federico II University Hospital, Naples, Italy

**Keywords:** liver cancer, hepatocarcinoma, radiofrequency ablation (RFA), colorectal cancer liver metastases (CRLM), cholangiocarcinoma (CC), liver surgery and transplantation, SBRT (stereotactic body radiation therapy)

The multidisciplinary evaluation and management is an established key factor for granting patients affected by liver tumours, whether primary or metastases, the best possible care. A holistic view and understanding are now mandatory, with the patient’s general conditions and wishes, oncological status, and therapeutic options, contributing simultaneously to create a personalised, precision-medicine approach. This is crucial in liver cancer patients, where evidence-based risks and benefits of the available surgical, radiological and oncological treatments need to be weighed against each other to select the best single or combined strategy. This Research Topic focuses on the efficacy of the radiofrequency ablation therapies alone or combined with other therapies in the treatment of primary and metastatic liver tumours.

The hepatocellular carcinoma (HCC) is the most frequent primary liver tumour and the one most frequently treated with ablation therapies. In the 2022 Barcelona Clinic Liver Cancer update (1), radiofrequency ablation (RFA) expands its possible roles in the treatment of Very early (0) and Early stage (A) HCC patients, as well as in Intermediate stages (B) as a downstaging therapy before liver transplantation (LT). Liver resection (LR) remains the treatment of choice, when feasible, for stage 0-A patients, providing superior overall (OS) and disease-free survival (DFS) when compared to RFA alone or in combination with chemoembolization (TACE), as highlighted in the Bayesian network meta-analysis by Zhang et al. In the same categories of patients, Jiang et al. confirmed these findings but identified a subgroup (age >65 years and tumour size ≤20 mm) in which RFA seems to grant survival results equal to the ones following LR. The availability of valid treatment options alternative to surgery is of paramount importance in patients unwilling or unfit to undergo procedures burdened by a high risk of morbidity and mortality such as LT or LR, even if technically resectable (2). The combination of RFA with other non-surgical techniques such as TACE has been explored in order to pursue the goal of maximising the oncological results, and consequently patient’s outcomes, but minimising the risk of severe complications that could jeopardise the quality of life. RFA combined with TACE has shown promising results in ensuring similar 1-, 3- and 5-year disease-free survival (DFS) when compared to LR, while granting significantly shorter hospital length of stay and a reduced incidence of major complications, as described by Dan et al. in their meta-analysis. In order to assess and quantify the risk of major complications following thermal ablations of primary and metastatic liver tumours, Huang et al. analysed the results of over 2000 consecutive procedures, detecting a thermal ablation-related mortality of only 0.1%, and an overall major complication rate of 5.6%, with pleural effusion being the most frequent complication as observed in the 2.9% of cases. They identified intrahepatic cholangiocarcinoma, a total maximum diameter of the lesions >3 cm, a low platelet count, the ablation performed with microwaves alone or in combination with RFA and the development of systemic inflammatory response syndrome, as independent risk factors for major complications at the multivariate logistic regression analyses. The limitations of the oncological efficacy of ablative techniques seem to be related to the size and the number of tumours, as shown in the retrospective analysis of Yue and Zhou, where over 2200 patients affected by multifocal HCC and treated with LR or RFA were compared through propensity score matching. Surgery confirmed to be the treatment of choice in such patients, being able to grant superior disease-specific and overall survival. Their work confirmed how the differentiation grade, alpha-fetoprotein, tumour size and extension were independent predictors of poor prognosis.

Organ shortage and patient’s fitness can limit the access of HCC patients to LT and LR, preventing them from receiving the ideal treatment. Alternative treatment options have been explored in these patients, and despite not holding the same curative potential, still have the chance to contrast tumour progression, and therefore prolong the survival or reduce the risk of drop-out from the transplant waiting list. The efficacy of Stereotactic Body Radiotherapy (SBRT) and RFA in HCC patients have been compared in a meta-analysis by Pan et al. of 10 retrospective studies. SBRT resulted to provide a better 1- and 3-year local tumour control, but conversely an inferior overall survival, possibly because of the better general conditions, smaller tumours and better liver function of the patients receiving RFA. Both treatments appeared to provide similar results when performed as a bridge therapy to LT.

The radiofrequency liver ablation is establishing its role in the complex scenario of liver tumour treatment thanks to the implementation of novel technologies, as well as the increasing experience in the combination with other local tumour control strategies and better case selection. The evidence regarding which groups of patients can benefit most from RFA is consolidating, and the minimal mortality and low morbidity are granting good tumour control results. Surgery and liver transplantation still provide better survival outcomes in HCC patients, but RFA represents a valid option especially in patients unfit for surgery, not meeting the criteria for LT or as a downstaging strategy.

## Author contributions

The author confirms being the sole contributor of this work and has approved it for publication.
